# A Model to Predict Heartbeat Rate Using Deep Learning Algorithms

**DOI:** 10.3390/healthcare11030330

**Published:** 2023-01-22

**Authors:** Ahmed Alsheikhy, Yahia F. Said, Tawfeeq Shawly, Husam Lahza

**Affiliations:** 1 Department of Electrical Engineering, College of Engineering, Northern Border University, Arar 91431, Saudi Arabia; 2 Department of Electrical Engineering, Faculty of Engineering at Rabigh, King Abdulaziz University, Jeddah 21589, Saudi Arabia; 3 Department of Information Technology, Faculty of Computing and Information Technology, King Abdulaziz University, Jeddah 21589, Saudi Arabia

**Keywords:** heartbeat, DCNN, LSTMs, artificial intelligence, face recognition, heart rate, cardiology, cardiovascular, MAE, MSE, ResNet50V2

## Abstract

ECG provides critical information in a waveform about the heart’s condition. This information is crucial to physicians as it is the first thing to be performed by cardiologists. When COVID-19 spread globally and became a pandemic, the government of Saudi Arabia placed various restrictions and guidelines to protect and save citizens and residents. One of these restrictions was preventing individuals from touching any surface in public and private places. In addition, the authorities placed a mandatory rule in all public facilities and the private sector to evaluate the temperature of individuals before entering. Thus, the idea of this study stems from the need to have a touchless technique to determine heartbeat rate. This article proposes a viable and dependable method to estimate an average heartbeat rate based on the reflected light on the skin. This model uses various deep learning tools, including AlexNet, Convolutional Neural Networks (CNNs), Long Short-Term Memory Networks (LSTMs), and ResNet50V2. Three scenarios have been conducted to evaluate and validate the presented model. In addition, the proposed approach takes its inputs from video streams and converts these streams into frames and images. Numerous trials have been conducted on volunteers to validate the method and assess its outputs in terms of accuracy, mean absolute error (MAE), and mean squared error (MSE). The proposed model achieves an average 99.78% accuracy, MAE is 0.142 when combing LSTMs and ResNet50V2, while MSE is 1.82. Moreover, a comparative measurement between the presented algorithm and some studies from the literature based on utilized methods, MAE, and MSE are performed. The achieved outcomes reveal that the developed technique surpasses other methods. Moreover, the findings show that this algorithm can be applied in healthcare facilities and aid physicians.

## 1. Introduction

Heartbeat measurement provides vital information to physicians or healthcare providers [1,2]. This procedure is the first activity to be performed by cardiologists once patients enter examining rooms. Cardiologists can quickly notice an issue with a patient if the measurement indicates that the heart rate is either high or low. Cardiologists use equipment or the traditional method to measure the heart rate, including touching hands. Therefore, there is a need to have a touchless technique to measure the heartbeat, and this need inspired the authors to conduct this study. This method should be reliable and provides an accurate result. 

Most of the mortality and economic burden are caused by cardiovascular diseases, and heart failure is the leading cause of mortality [1]. More than 50% of mortality is caused by cardiovascular diseases [1,2,3,4]. Due to technological advancements that involve monitoring and sensor systems, it has become easy to measure the heartbeat. However, this measurement requires skin contact [1]. Physicians use the traditional method to measure the heartbeat or the ECG signal. ECG stands for electrocardiogram, a graphical representation of cardiac activities [1,2,3]. These activities are captured by placing adhesive electrodes on the body’s surface. These electrodes sense any small electrical changes due to cardiac muscle movements caused by repolarization and depolarization during every cardiac cycle [2,3,4,5].

ECG represents the cardiac function characterized and demonstrated by the correlation between peaks and phases. Any regular cardiac activity of heartbeat occurs within P, QRS, and T waves [6]. A typical ECG waveform contains a P wave, PR wave, QRS complex, ST segment wave, T wave, and U wave. The atrial depolarization is represented by the P wave [3,4,5,6,7,8]. QRS complex wave denotes ventricular depolarization [6,7,8]. The T wave expresses ventricular repolarization, while the U wave indicates papillose muscle repolarization [6,7,8]. 

### 1.1. Research Problem

Various contactless systems and applications have been implemented to measure heartbeat rates continually. These techniques rely on sensors to determine the heart rate [9,10]. In addition, mobility is not a concern since these techniques require no constraints or boundaries [11,12,13,14]. Individuals who suffer from skin irritations can utilize these techniques freely. These systems deliver real-time data and results for heartbeats as users can check their heart rate continuously. Cardiologists and patients have confidence in these approaches to identify unusual or irregular cardiac activities [14]. These unique or rare activities might lead to difficult situations and illnesses such as tiredness, dizziness, and shakiness [14,15,16,17].

Physicians utilize the adaptability and flexibility of the heart rate for several objectives, such as illness confirmation and stress recognition [18,19,20,21,22,23,24,25,26]. In addition, these two factors characterize symptoms of any existing heart disease, such as high blood pressure or coronary disease [17,18,19].

### 1.2. Motivations and Contributions

Object and pattern recognition methods, such as computer vision (CV), are utilized in various fields to identify objects or targets, such as faces. The Viola–Jones approach is used to detect faces. This method was developed in 2001 and named by its inventors, Paul Viola and Michael Jones. This algorithm is a helpful tool for face identification due to its accuracy. This study uses the proposed algorithm to capture and detect hands. The proposed technique predicts the average number of heartbeats from the detected hands. This approach extracts mean, median, variance, and standard deviation features from a motion-based detection algorithm and other statistical characteristics. It delivers two outputs, a graphical representation of peaks and bottoms of the required features and the estimated heartbeat rate.

Implementing a touchless methodology to plot a graphical waveform representation of the extracted features to calculate the cardiac rate is the inspiration in this study due to the restrictions of the COVID-19 pandemic; this model supports and assists cardiologists in diagnosing heart rate. In addition, predicting whether there is a potential heart disease is another motivation for performing this research. 

The contribution herein is offered by implementing and suggesting a contactless algorithm to predict the heartbeat and tell whether a person suffers from potential heart diseases by indicating whether their heart rates are low or high. The rest of the paper is structured as follows: a literature review is given in the next section, and Section 3 details the proposed approach. Results are in Section 4, followed by a discussion in Section 5, and the conclusion is in Section 6.

## 2. Related Work

A. K. S. Saranya and T. Jaya in [1] developed a heartbeat rate detector based on a Krill Deep Neural Network Stacked Auto Encoders (KDNN-SAE) to predict heart rate using multimodal data. The purpose of the developed method was to classify cardiac diseases by combining multiple ECG signals. An adaptive filter enthalpy-based Empirical Model Decomposition (EMD) was included in the training stage to eliminate the motion artifact in the considered signals. The extracted values of features were normalized to estimate the loss function. This function was calculated by Robotic Process Automation (RPA). The authors claimed that their method achieved 99.17% accuracy. In this study, the proposed approach extracts features of various factors and determines the average value for each feature. Finding the maximum average value between all features is performed. Later, the mean of the corresponding feature is computed to estimate the average value of the heartbeat by using its corresponding time. This method requires only a filter to remove noise and works based on the motion, not a regular image. This method achieved 99.78% accuracy on volunteers. Moreover, the calculated MAE and MSE were ±0.142 and ±1.82, respectively. The proposed algorithm in this research achieves better results than the developed method in [1]. Readers can refer to [1] for additional information. 

E. S. Pramukantoro and A. Gofuku in [2] presented a method to classify heartbeat in real-time according to RR interval data obtained from a wearable device. The authors used machine learning and deep learning techniques to train multiple classifiers. The authors evaluated their method based on several performance metrics: accuracy, precision, recall, and F-score. The implemented approach achieved an average of 96.103% accuracy for all utilized classifiers, while the maximum achieved accuracy was 96.67% for a random forest classifier. In addition, that approach used nine features, while the proposed model in this study utilized 14 features. The presented algorithm in this article uses three deep learning tools to train and validate its results on volunteer data. This method achieved an average 99.78% accuracy, which is better than the method in [2], and the maximum obtained accuracy was 99.88%. Moreover, the technique developed in [2] worked based on the ECG signal, which requires contact on individuals’ bodies, while the presented approach in this research requires no connection as it is a touchless method.

In [4], A. Staffini et al. compared different forecasting methods: Autoregressive, Long Short-Term Memory Network, and Convolutional Long Short-Term Memory Network. The authors performed the analysis on twelve participants. The collected data were obtained using a wearable device for more than ten days. The autoregressive model was the best as it produced the best results among other models. The proposed model obtains data from heterogeneous volunteers in current health status, types of movements during the conducting experiments, age, sex, and past medical history. Moreover, this approach determines the heart rate instantly, which is faster than the developed method in [4]. The obtained average accuracy in the presented algorithm was 99.78% when integrating ResNet50V2 and LSTMs, while the authors in [4] provide no information about the accuracy of their approach. Interested readers can refer to [4] for additional information.

M. Oyeleye et al. in [7] explored multiple classifiers to predict heart rate. These classifiers were Autoregressive Integrated Moving Average (ARIMA), Linear Regression, Support Vector Regression (SVR), K-Nearest Neighbor Regressor, Decision Regressor, and Random Forest Regressor. The authors utilized these models to analyze the accelerometer data to perform future predictions. The analysis lasted 30s to 1 h to determine MAE, MSE, RMSE, and scattered index. All classifiers produced different values for both tests, and the minimum MAE was from ARIMA. The authors should have provided more information about accuracy. The proposed model herein requires no time window to predict the heartbeat rate as it determines it instantly. The obtained accuracy was 99.78%, while other considered performance metrics were ±0.142 and ±1.82 for MAE and MSE during the testing stage, respectively, as proved by the conducted experiments described in Section 4. 

N. Homdee et al. in [14] developed a feasible method to estimate raw heart rate based on obtained data from a smartphone using its sensors. The authors collected data from twelve participants during a week-long study period. Three different models to classify data were utilized: Regression, SVM, and Random Forest. The authors found that SVM yielded the minimum RMSE values among other models. However, no information about accuracy was provided by the authors. On the other hand, the proposed model in this study uses various deep learning techniques (DLTs) to estimate the heart rate based on computing the average values of all considered features and obtaining the maximum average value among the calculated values. Later, this figure is utilized to determine the mean of the corresponding feature and use it to predict the heartbeat pulse. Moreover, this algorithm achieves 99.78% average accuracy and outperforms other works in accuracy and other considered metrics.

## 3. Materials and Methods

### 3.1. Problem Statement

Nowadays, various methods have been implemented to predict heartbeat. These approaches focus on using sensors to collect data. These methods are portable but not contactless since the utilized sensors are required to be placed on the skin to obtain data for heart rate prediction. This study emphasizes developing a contactless algorithm to estimate the heartbeat rate precisely. In this research, contactless means there is no need for wires to be placed on the body’s surface or for any sensor to touch the skin to collect data. Only video streams are required to estimate the heart pulse. Consequently, the authors aim to implement a practical, reliable, and touchless methodology to predict heart rate more accurately than what has been reached. Moreover, indicating whether people have potential heart diseases is considered in this study as well. This is achieved automatically by the proposed model by comparing the estimated heartbeat rates with regular average heart pulses.

### 3.2. Research Objectives

To bring up the proposed method into reality, the objectives of this research are:

1. A reliable technique to appropriately predict the average number of heartbeats;
2. This technique should be completely contactless;
3. Obtained accuracy shall be more than what has been reached from the recently implemented models;
4. The developed technique shall support cardiologists by providing a quick overview of patients’ conditions and telling whether these patients have cardiac issues;
5. Ability to identify any health issues from the heart rate estimation since low or high values indicate these problems;
6. Alert cardiologists once the low or high heartbeat is estimated;
7. Determine the required performance metrics: accuracy, MAE, and MSE;
8. Assess the proposed technique and other state-of-the-art works from the literature by comparing the achieved findings of MAE and MSE.

### 3.3. Utilized Deep Learning Techniques

#### 3.3.1. AlexNet

**Figure 1:**
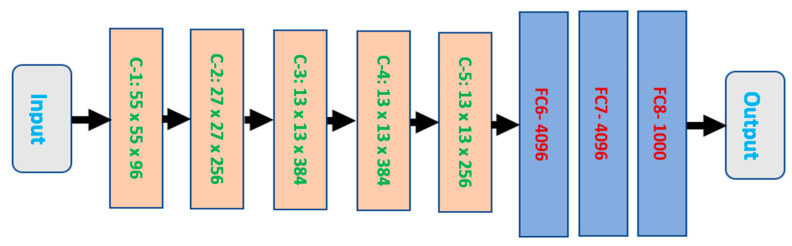
A typical AlexNet structure.

AlexNet was developed in 2012 by two researchers and was among the top five winners. AlexNet contains eight different layers between the first and the last, which are the input and the output, as illustrated in Figure 1. Each layer between the input and the output layers is associated with its size and number of neurons. C-X in the first five layers stands for convolutional, X refers to the layer number, while FC stands for the fully connected layers. All eight layers have numerous learnable parameters, and the ReLU activation function is used except in the last layer. This tool accepts inputs of size 227 × 227 to process them. This size is converted to different sizes, as depicted in Figure 1. AlexNet utilizes numerous filters, such as 11 × 11, 5 × 5, and 3 × 3. AlexNet takes inputs as RGB images. Hence, every video frame is converted into RGB images. All required parameters of the DCNNs are shown in Figure 1.

#### 3.3.2. ResNet50V2

ResNet50 is another convolutional neural network that was developed in 2016. Microsoft implemented ResNet50 with 96.4% accuracy. Its depth is up to 152 layers. Due to space limitations, the ResNet50 structure is omitted. ResNet50V2 was developed in 2020 and is a recently developed deep learning technique. This model is customized in this research to be integrated with AlexNet to reach a high level of accuracy by using 4 blocks.

#### 3.3.3. Long Short-Term Memory Networks (LSTMs)

LSTMs are a type of deep learning technique utilized in artificial intelligence. Specifically, LSTMs are a Recurrent Neural Network (RNN) type. This tool is customized and used in this study alone. In addition, integration based on the concatenation between LSTMs and ResNet50V2 is performed to generate better accuracy.

### 3.4. The Proposed Methodology

In this study, a real-time model to estimate the average heartbeat rates and to indicate if there is a heart issue is developed. The presented approach shall generate its outputs before receiving another input. This study considers all possible activities, such as running, walking, and playing. The proposed methodology works by computing the average values of all extracted features. After that, the average for every feature is calculated, and a maximum average value is selected. Then, the two top values of the corresponding feature chosen are taken, and their mean is computed. Later, the corresponding time is utilized to determine the average heartbeat rate. The demonstration of the presented model is conducted with several random participants from two locations. 

This subsection provides details and a complete description of the presented model. This model predicts the heartbeat rate with high accuracy. It utilizes two Deep Convolutional Neural Network (DCNN) tools, AlexNet and ResNet50V2, and a deep learning technique, LSTMs, to train and test the proposed algorithm. Several schemes are performed in this study to achieve high results and compare them. These schemes are presented in Section 4. The given model involves various stages to reach its optimal goals: accurately estimating the heart pulse and assisting physicians by telling whether a patient suffers from a cardiac issue. The volunteers who participated in this research were heterogeneous, as shown in Table 1. Moreover, none of the participants drank alcohol or smoked. All volunteers were in a healthy condition as no history was disclosed except for a child diagnosed with a global development delay. This child is referred to as number nine in the table. These volunteers were collected in two days in two locations, and the number of volunteers is small; this is a drawback and a limitation since the authors could not obtain more volunteers. In addition, the volunteers requested to keep their identities private to participate. The age of volunteers ranged from 6 years old to 44 years old.

The quality of inputs is enhanced in the proposed system by using image preprocessing methods to extract insightful features. These features are variance, mean, median, standard deviation, correlation, Chi-square, RMSE, regression, Wilcoxon’s signed rank test, Mann–Whitney test, Kolmogorov–Smirnov test, Kruskal–Wallis test, Jonckeere test, and Friedman test. The implemented technique was tested on the 5-Fold Cross-Validation method in this study, as illustrated in Table 2. The mean age of participants was 20.89 years old, and the standard deviation was 16.42 years old. 

Volunteers in this study were asked to participate at one of the biggest shopping centers in Jeddah, Saudi Arabia, while the second location was in another city. This city is Arar, Saudi Arabia. These volunteers participated in the second week of October 2022 and the third week of December 2022 between 10 am and 4 pm. Furthermore, we focused our analysis and measurements on activities impacting heart pulses, such as performing exercises or cleaning. However, one volunteer was sitting on a couch during the experiments.

**Figure 2:**
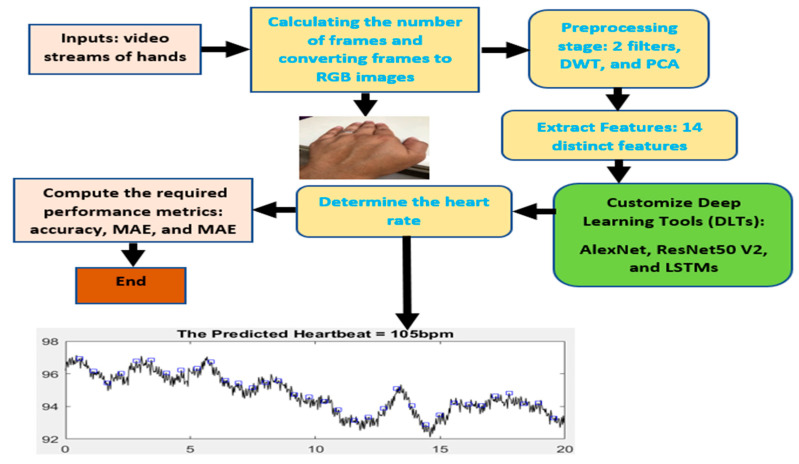
The flowchart of the proposed technique.

The presented model is an ensemble of three deep learning techniques: two convolutional neural networks (DCNNs): AlexNet and ResNet50V2, and LSTMs, as depicted in Figure 2. These three techniques were chosen according to their accuracy, easiness, performance in feature selection, and fastness. Adam optimizer is utilized in this study, and a batch normalization approach is also included. Four stacked layers were implemented herein to carry out the customization and integration of ResNet50V2 and LSTMs. The first layer contains 25 cells; the remaining layers have 50 cells. In addition, tanh is utilized as the activation function inside LSTMs. This developed architecture of ResNet50V2 and LSTMs was achieved after several experiments.

The proposed approach starts by taking images of captured video streams. Then, the algorithm determines the number of frames in the video streams and converts these frames to RGB images. The preprocessing and segmentation stage is performed based on the measured number of frames to rescale these inputs to the acceptable size of AlexNet and ResNet50V2, remove noise through two built-in filters, and enhance the quality of images. Then, hand recognition from video frames is applied to extract the required features. In addition, finding the mean value of every feature is performed. The proposed technique determines the maximum mean value to calculate the corresponding mean of the two top peaks. This value is used to predict the average value of the heartbeat rate by taking its corresponding time. 

Initially, the implemented Algorithm 1 assumes that the initial heartbeat rate is 60 bpm, the minimum standard value used by cardiologists. Some utilized equations are from [27]. Figure 2 illustrates a block diagram of the proposed model with input and output. Quantitative and qualitative findings are obtained from accuracy, MSE, and MAE. Additionally, deep analysis and investigation of using a further regression stage to improve the heartbeat rate prediction from volunteers are performed.

The proposed technique delivers various features and advantages, which are summarized as follows:

- Completely contactless model;
- Portable model. Applicable anywhere and anytime;
- No special equipment is required.

The following pseudo-codes determine how the proposed model is implemented:
**Algorithm 1: Contactless Heartbeat rate prediction****Input: a video stream** **Output: instant average heartbeat rate (bpm) prediction. **Read a video stream from a camera or a file.Determine the number of frames from the video file, a.Covert frames to RGB images.Detect faces using the Viola–Jones method and put rectangular boxes around the detecting faces.Image segmentation: rescale images, remove noise, enhance the quality, and convert to grayscale.Create zeros matrices for two variables, b and c, where b refers to the images captured from the frames and c represents the equivalent images after converting into grayscale.**For (i = 1: a)**   Extract the required features and save their results in d(i)**End**Find the maximum value of all computed features of the matrix, d.For the feature with the maximum value, determine its mean for the two highest numbers and save it in M.Initialize several parameters, t_p_, counter, and hp, to predict the heartbeat where t_p_ refers to the last time, counter with initial value = 0. H_p_ denotes the initial heart rate of 60 bpm.**For (i = 1: a)**   Read the current time (t) from the source file.   Predict the heartbeat rate and save the result in h_n_. Initially, h_n =_ h_p._   h_n_ = [ (h_n_ * (counter+1))/((t- t_p_) * 60) ] / [ counter +1]   Ceil the result in h_n_.   Increment the counter.   Set the last time, t_p_, to be the current time, t.**End****Calculate accuracy, MAE, and MSE.**End of the algorithm.

## 4. Results

Several trials were performed on MATLAB to verify the developed algorithm and examine its procedures and outcomes. The model was running over 300 times, and it took approximately 47mins for the training period to finish and achieve the desired results. During the training stage, the utilized loops were running 1000 times to optimize the results, increase accuracy, and minimize MAE and MSE. The computations of the required performance metrics are shown in this section. In addition, a comparison measurement between some developed works from the literature and the proposed technique is performed. MATLAB is utilized in this study due to its powerfulness in dealing with images and the availability of built-in toolboxes. MATLAB is installed on a machine that runs with Windows 11 Pro. This device has a specification; which is an Intel Core i7-85550U with a clock speed of 2GHz. The size of the installed RAM is 16GB. During the experiments, the following parameters were set, which are as follows:

- 10 fully connected layers (FC) were used in AlexNet;
- The maximum number of utilized blocks in ResNet50V2 is 4;
- The learning rate (L) changes between 0.01 and 0.0001;
- The number of maximum epochs was between 8, 10, and 62.

The performance calculation is quantitative and qualitative of the obtained accuracy, MAE and MSE, based on three scenarios mentioned in Section 3.3.2 and Section 3.3.3. The implemented technique evaluates the metrics on 20 volunteers; the average rate is taken for every metric. The size of participants is small, which is a drawback as many individuals refused to participate in this study even though the authors provided some promotions to stimulate them to agree and be part of this research.

The predicted value is compared with the actual heartbeat measured by a portable device from a pharmacy, as shown in the coming Section 4.1, Section 4.2 and Section 4.3. The used portable device is called Omron M6 Comfort. 

### 4.1. Customization and Integration of AlexNet and ResNet50V2

**Figure 3:**
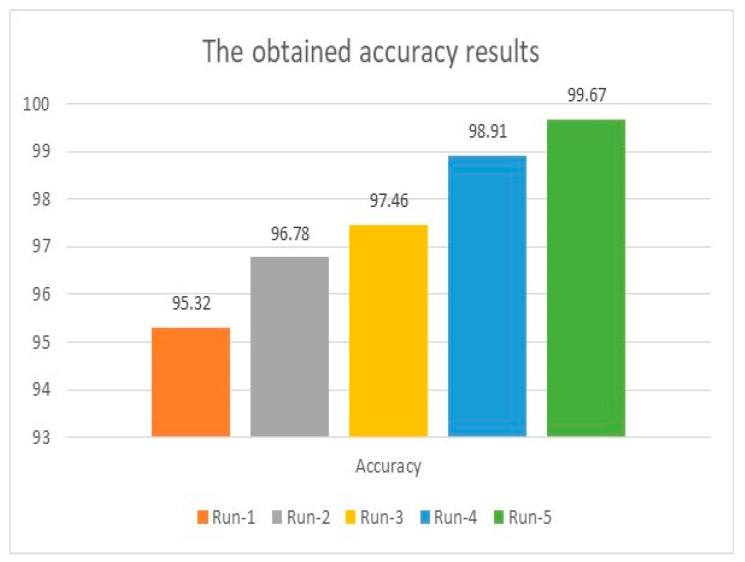
Accuracy analysis using the integration of AlexNet and ResNet50V2.

**Figure 4:**
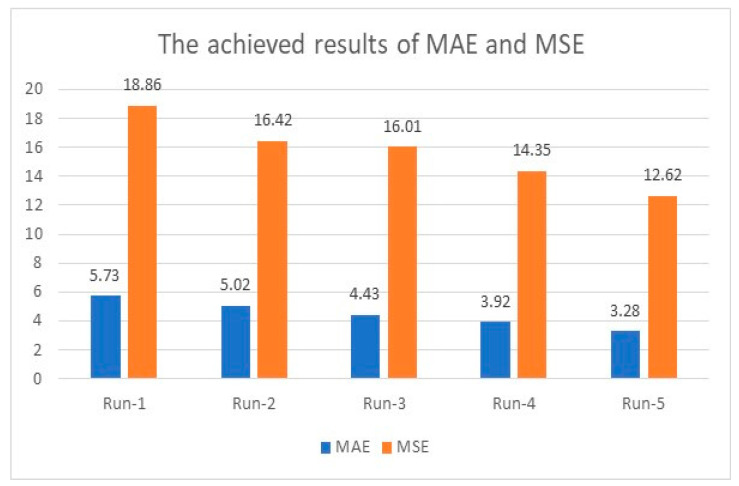
MAE and MSE evaluation using the integration of AlexNet and ResNet50V2.

The initial accuracy was 95.32% when the model ran for 200 iterations and improved as the number of distinct runs increased and reached a maximum accuracy of 99.81%. For instance, in the first distinct run, the presented algorithm reached 95.32%, 5.73, and 18.86 for accuracy, MAE, and MSE. These figures enhanced as the number of distinct runs increased, as depicted in Figure 3 and Figure 4. These two graphs show that all considered measurements improved after each distinct run and reached an appropriate level of 99.67% or more, as the maximum obtained accuracy was 99.81%.

In Figure 3, the accuracy improved at a noticeable pace and increased rapidly to reach 99.67% in the fifth distinct run. In Figure 4, The change of MAE values was apparent and in a good step, while MSE changed faster than MAE and obtained a minimum value of 12.62.

Table 3 lists the actual heartbeat, predicted heart rate, and error percentage between actual and estimated heart rates. This table indicates that the maximum obtained error among all volunteers is 3.125%. In addition, this table shows that the proposed technique correctly predicted 70% of the sample size. Moreover, the obtained MAE was 0.3, and MSE was 1.8. Both values are small, implying that the presented model estimated heart rate with an acceptable level of correctness. To obtain the actual heartbeat rates for all participants, the portable electronic device from the pharmacy was used, and this procedure was performed once for all volunteers. The presented model ran 1000 times for the prediction stage, and the mean was computed. 

**Figure 5:**
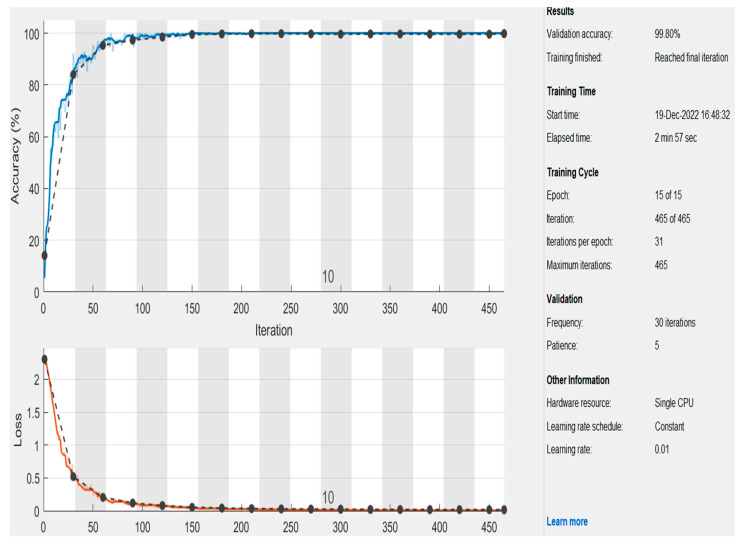
The achieved results of accuracy and the loss function using L_a_.

Figure 5 depicts the obtained accuracy when the learning rate (L_a_) was 0.01, and the number of epochs was 15. The black dashed line represents the validation stage, while the blue line refers to the obtained accuracy. This graph shows that the presented technique reached 99.80% accuracy and the loss function converged to almost zero for 465 iterations. In addition, the proposed model obtains higher precision when the learning rate parameter is significant. It requires a smaller number of iterations and epochs to achieve high accuracy.

**Figure 6:**
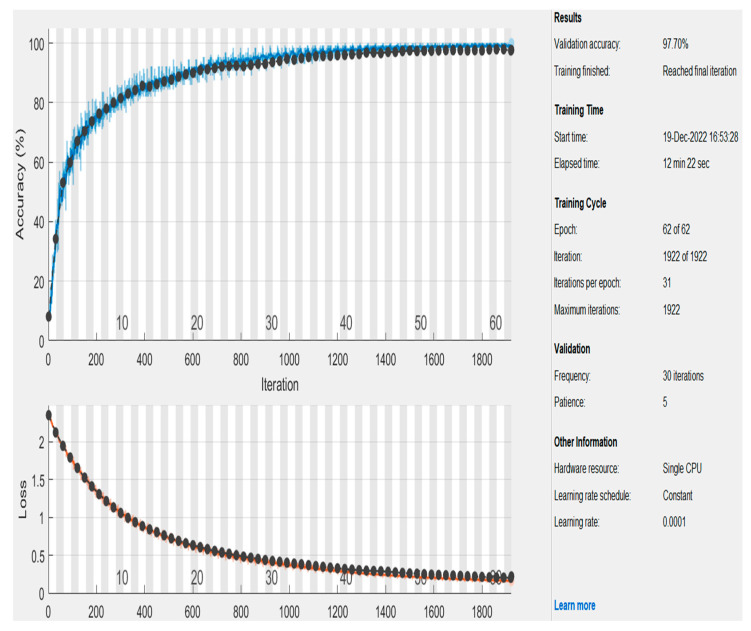
The accomplished graphs of accuracy and the loss function using L_b_.

Figure 6 illustrates the obtained accuracy and loss function when the learning rate parameter (L_b_) was 0.0001. The model reached 97.70% accuracy when the number of epochs was 62 and the number of iterations was 1922. This is obvious as the proposed model requires more time to learn deeply since the learning rate parameter was small.

### 4.2. LSTMs

**Figure 7:**
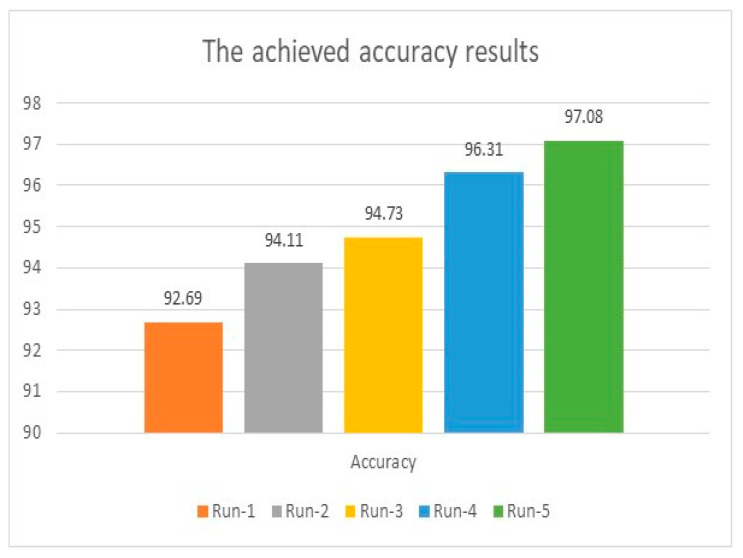
Accuracy evaluation using LSTMs model.

**Figure 8:**
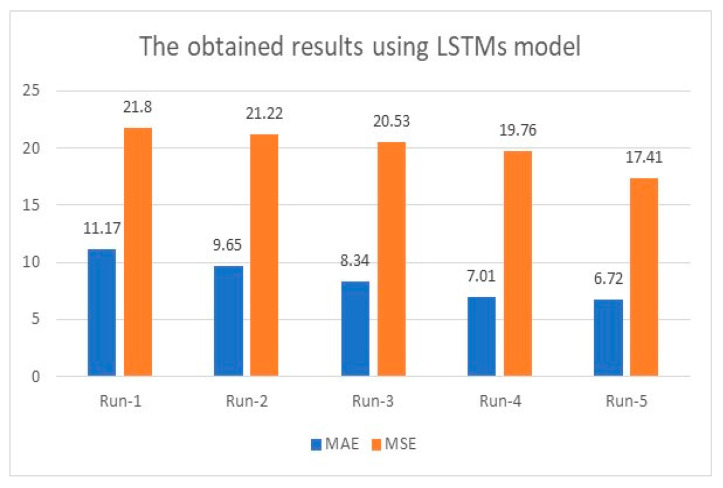
MAE and MSE evaluation using LSTMs tool.

The initial accuracy was 92.69% when the model ran for the same number of iterations conducted in Section 4.1, which was 200 iterations. The accuracy was enhanced as the number of distinct runs increased and reached a maximum accuracy of 98.3%. For instance, in the first distinct run, the presented algorithm reached 92.69%, 11.17, and 21.8 for accuracy, MAE, and MSE. These figures were enhanced as the number of distinct runs increased, as depicted in Figure 7 and Figure 8. The results of accuracy, MAE, and MSE using LSTMs indicate that LSTMs produced good results. However, these results are smaller than what has been reached when combining AlexNet and ResNet50V2, as illustrated in Figure 3 and Figure 4.

This technique reached 97.64% accuracy when the learning rate was 0.01 and 97.9% with a 0.0001 learning rate when the number of epochs was 62. In addition, the LSTMs technique predicted eight samples accurately, which means 57.143% of the sample size. Moreover, the obtained MAE was 5.82, and MSE was 14.33 after 12 distinct runs. Both values of MAE and MSE are bigger than what is achieved in Figure 4 and indicate that utilizing LSTMs alone is insufficient.

### 4.3. Concatenation of LSTMs and ResNet50V2

**Figure 9:**
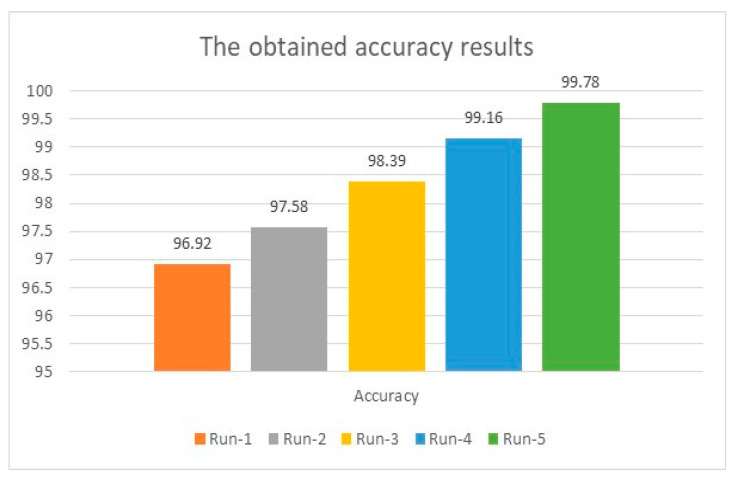
Accuracy analysis using customized LSTMs and ResNet50V2.

**Figure 10:**
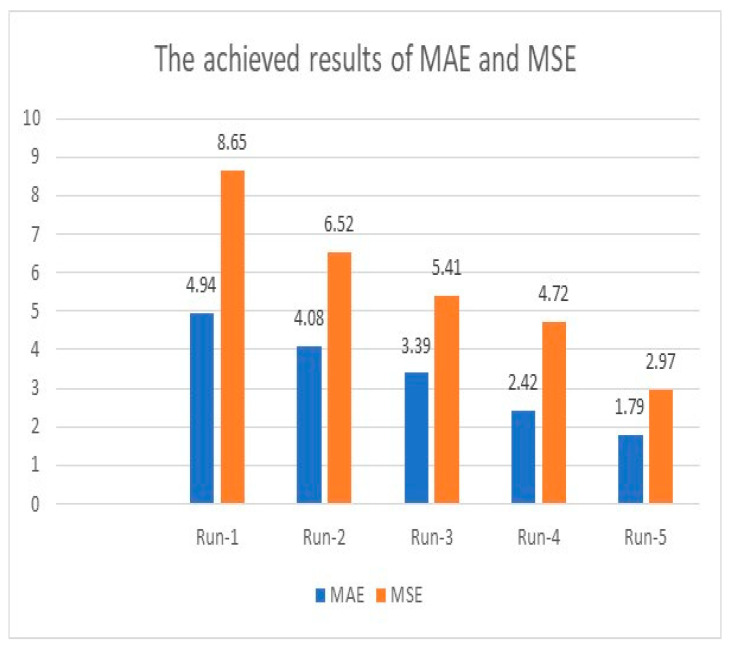
MAE and MSE analysis using the integrated deep learning approaches.

Initially, the achieved accuracy was 96.92% when the model ran for the same number of iterations conducted in the previous two subsections. The accuracy was boosted as the number of distinct runs increased and reached a maximum accuracy of 99.88%. For instance, in the first distinct run, the presented algorithm earned 96.92%, 4.94, and 8.65 for accuracy, MAE, and MSE. These values improved as the number of distinct runs increased, as illustrated in Figure 9 and Figure 10. The results of accuracy, MAE, and MSE using customized and integrated LSTMs and ResNet50V2 indicate that these customizations and integrations produce exquisite findings. Furthermore, this algorithm estimated 12 samples correctly out of the total sample size of 85.71%. This value is the highest value reached in this research. Moreover, the obtained MAE was 0.142, whereas MSE was 1.82 after seven distinct runs. The obtained average accuracy of 99.78% is the highest reached value. The maximum achieved accuracy is 99.88%. Both values of MAE and MSE are the most minor obtained outcomes. Table 4 displays the obtained results using LSTMs and ResNet50V2 together.

The comparative evaluation between the developed technique in this study and other implemented approaches in the literature is conducted according to the methodologies being utilized, the obtained values of MAE, MSE, and a type of these models, whether contactless or not, which are shown in Table 5. The results of the presented system refer to the use of LSTMs and ResNet50V2. N.M. in the same table stands for not mentioned.

## 5. Discussion

**Figure 11:**
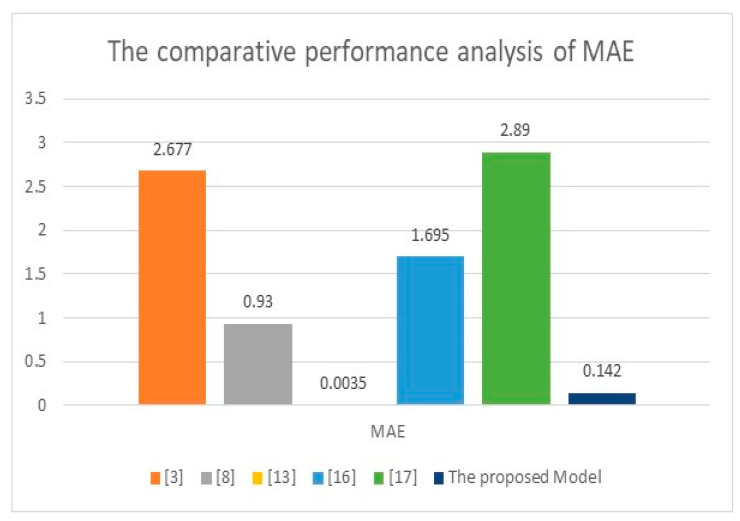
The comparative evaluation of the achieved MAE with different implemented methods [3,8,13,16,17].

**Figure 12:**
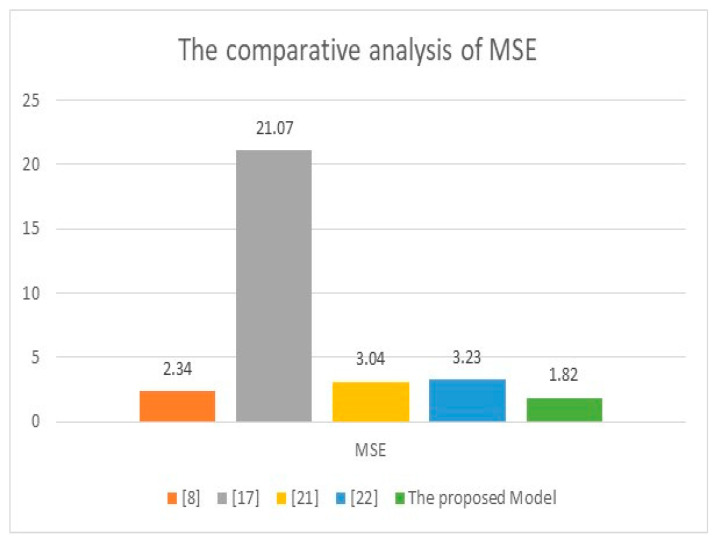
The comparative analysis of MSE between the presented model and some developed algorithms [8,17,21,22].

Table 5 shows that all the previously developed models required physical contact to predict the heartbeat. In contrast, the proposed model herein requires no physical contact since the prediction is based on information from video streams. The implemented model in [13] obtained the smallest MAE since it was applied to 1025 cases, whereas the presented technique in this study tested only 20 participants and achieved 0.142. Figure 11 and Figure 12 illustrate the comparative performance analysis between the proposed method and some works from the literature on MAE and MSE. In Figure 11, the maximum obtained MAEs were in [3,16,17]. The highest MAEs were 2.89, 2.677, and 1.695. The developed model in [8] achieved a moderate MAE of 0.93. In contrast, the presented algorithm in this research achieved 0.142, the minimum obtained value of MAE, except for the implemented model in [13], which reached 0.0035 due to the data sample size, which was 1025. In contrast, the proposed model was applied to 20 volunteers. In Figure 12, the developed model in [17] achieved the highest value of MSE, 21.07. In contrast, the model in [8] performed a moderate result of MSE, which was 2.34, while the presented technique reached the minimum MSE value of 1.82. Both graphs show that the proposed approach outperformed other works implemented earlier and achieved the minimum values of MAE and MSE except the developed method in [13] for MAE. 

The proposed model can be installed on a regular hosting machine with an installed camera or on a special device equipped with artificial intelligence (AI) software. The first option is cheaper than the second one, as special machines are costly. 

During the conducted analysis, the movement of hands was not included or considered, as it will be included along with the gesture in future work.

## 6. Conclusions

This research proposes a novel method to predict heartbeat rate using a contactless technique. The presented model utilizes numerous deep learning tools to train the model and provide a test on 20 participants. The obtained results show that the proposed model adequately predicts the heart rate. The average achieved accuracy was 99.78%, and the maximum was 99.88%. These results are exquisite and can be applied anywhere and anytime without needing equipment or special hardware. Cardiologists can depend on this algorithm to estimate patients’ heart rates upon entering the examination rooms. In addition, this model can predict whether a patient suffers from cardiac issues since a low or a high heartbeat implies that a problem or a condition occurred. This model needs more time to process data as numerous processes are already involved.

Predicting the heart rates from individuals’ eyes, the movement of hands and gestures will be considered in future work. Moreover, other performance metrics, such as precision, recall, specificity, and F-score, will also be considered.

## Figures and Tables

**Table 1:** Characteristics of volunteers.

Volunteer Number	Gender	Adult/ Minor	Medical History	Exercise Type
1	F	Adult	No	Jogging
2	M	Adult	No	Playing Football
3	M	Adult	No	Playing Football
4	M	Adult	No	Playing Football
5	F	Adult	No	Running
6	F	Minor	No	Sitting on a couch
7	F	Minor	No	Walking
8	F	Minor	No	Walking
9	F	Minor	Yes	Not Applicable
10	F	Adult	No	Cleaning
11	M	Minor	No	Playing Volleyball
12	M	Adult	No	Push Ups
13	M	Adult	No	Jumping
14	M	Minor	No	Walking
15	F	Adult	No	Cleaning
16	F	Adult	No	Walking
17	F	Minor	No	Playing Tennis
18	M	Minor	No	Running
19	M	Adult	No	Playing Basketball
20	M	Adult	No	Doing Excercise

**Table 2:** The scenario of 5-Fold cross-validation.

	Fold-1	Fold-2	Fold-3	Fold-4	Fold-5
**Run-1**	Test	Train	Train	Train	Train
**Run-2**	Train	Test	Train	Train	Train
**Run-3**	Train	Train	Test	Train	Train
**Run-4**	Train	Train	Train	Test	Train
**Run-5**	Train	Train	Train	Train	Test

**Table 3:** The results of the proposed model.

Volunteer Number	Actual bpm	Predicted bpm	Error %
1	72	72	0
2	92	92	0
3	95	95	0
4	78	78	0
5	102	101	0.980%
6	69	69	0
7	82	81	1.22%
8	76	76	0
9	62	62	0
10	67	67	0
11	89	89	0
12	96	95	1.041%
13	74	73	1.35%
14	84	84	0
15	71	71	0
16	64	62	3.125%
17	83	82	1.205%
18	93	93	0
19	81	81	0
20	78	78	0

**Table 4:** The results of the proposed model.

Volunteer Number	Actual bpm	Predicted bpm	Error %
1	72	72	0
2	92	92	0
3	95	94	1.052
4	78	78	0
5	102	102	0
6	69	69	0
7	82	82	0
8	76	76	0
9	62	60	3.226%
10	67	67	0
11	89	89	0
12	96	96	0
13	74	74	0
14	84	84	0
15	71	71	0
16	64	64	0
17	83	83	0
18	93	93	0
19	81	81	0
20	78	78	0

**Table 5:** The comparative analysis results.

Works	Model Being Used	MAE	MSE	Physical Contact or Contactless
[1], 2023	RPA and KDNN-SAE	N.M.	N.M.	Physical contact: requires ECG.
[2], 2022	RR interval, Polar H10, and different classifiers	N.M.	N.M.	Physical contact: requires a wearable sensor
[3], 2021	Autoregressive and CNN	2.677	N.M.	Physical contact: requires ECG
[8], 2022	ARIMA, SVR, KNN, LR, DT, RF, and LSTM	0.93	2.34	Physical contact: requires ECG
[13], 2020	Nine classifiers	0.0035	N.M.	Physical contact
[16], 2019	LSTM	1.695	N.M.	Physical contact
[17], 2018	An active learning	2.89	21.07	Physical contact
The proposed system	Various deep learning models (AlexNet, ResNet50V2, and LSTMs)	0.142	1.82	Contactless

## Data Availability

Not applicable.
